# Biomarkers to improve functional outcome prediction after ischemic stroke: Results from the SICFAIL, STRAWINSKI, and PREDICT studies

**DOI:** 10.1177/23969873241250272

**Published:** 2024-05-06

**Authors:** Felipe A Montellano, Viktoria Rücker, Kathrin Ungethüm, Anna Penalba, Benjamin Hotter, Marina Giralt, Silke Wiedmann, Daniel Mackenrodt, Caroline Morbach, Stefan Frantz, Stefan Störk, William N Whiteley, Christoph Kleinschnitz, Andreas Meisel, Joan Montaner, Karl Georg Haeusler, Peter U Heuschmann

**Affiliations:** 1 Institute of Clinical Epidemiology and Biometry, Julius-Maximilians-Universität (JMU) Würzburg, Würzburg, Germany; 2 Department of Neurology, University Hospital Würzburg, Würzburg, Germany; 3 Interdisciplinary Center for Clinical Research, University Hospital Würzburg, Würzburg, Germany; 4 Institute of Medical Data Science, University Hospital Würzburg, Würzburg, Germany; 5 Neurovascular Research Laboratory, Vall d’Hebron Institute of Research, Universitat Autònoma de Barcelona, Barcelona, Spain; 6 Department of Neurology and Experimental Neurology, Charité – Universitätsmedizin Berlin, Berlin, Germany; 7 Center for Stroke Research Berlin, Charité-Universitätsmedizin Berlin, Berlin, Germany; 8 NeuroCure Clinical Research Center, Charité-Universitätsmedizin Berlin, Berlin, Germany; 9 Department of Biochemistry, Vall d’Hebron University Hospital, Barcelona, Spain; 10 Charité-Universitätsmedizin Berlin, Corporate Member of Freie Universität Berlin and Humboldt Universität zu Berlin, Berlin, Germany; 11 Department Clinical Research & Epidemiology, Comprehensive Heart Failure Center, University Hospital Würzburg, Würzburg, Germany; 12 Department of Internal Medicine I, University Hospital Würzburg, Würzburg, Germany; 13 Centre for Clinical Brain Sciences, University of Edinburgh, Edinburgh, UK; 14 Department of Neurology and Center for Translational Neuroscience and Behavioural Science (C-TNBS), University Hospital Essen, Essen, Germany; 15 Stroke Research Program, Instituto de Biomedicina de Sevilla/Hospital Universitario Virgen del Rocío/Consejo Superior de Investigaciones Científicas/University of Seville, Seville, Spain; 16 Department of Neurology, Hospital Universitario Virgen Macarena, Seville, Spain; 17 Clinical Trial Center Würzburg, University Hospital Würzburg, Würzburg, Germany

**Keywords:** Prognosis, biomarkers, stroke

## Abstract

**Background and aims:**

Acute ischemic stroke (AIS) outcome prognostication remains challenging despite available prognostic models. We investigated whether a biomarker panel improves the predictive performance of established prognostic scores.

**Methods:**

We investigated the improvement in discrimination, calibration, and overall performance by adding five biomarkers (procalcitonin, copeptin, cortisol, mid-regional pro-atrial natriuretic peptide (MR-proANP), and N-terminal pro-B-type natriuretic peptide (NT-proBNP)) to the Acute Stroke Registry and Analysis of Lausanne (ASTRAL) and age/NIHSS scores using data from two prospective cohort studies (SICFAIL, PREDICT) and one clinical trial (STRAWINSKI). Poor outcome was defined as mRS > 2 at 12 (SICFAIL, derivation dataset) or 3 months (PREDICT/STRAWINSKI, pooled external validation dataset).

**Results:**

Among 412 SICFAIL participants (median age 70 years, quartiles 59–78; 63% male; median NIHSS score 3, quartiles 1–5), 29% had a poor outcome. Area under the curve of the ASTRAL and age/NIHSS were 0.76 (95% CI 0.71–0.81) and 0.77 (95% CI 0.73–0.82), respectively. Copeptin (0.79, 95% CI 0.74–0.84), NT-proBNP (0.80, 95% CI 0.76–0.84), and MR-proANP (0.79, 95% CI 0.75–0.84) significantly improved ASTRAL score’s discrimination, calibration, and overall performance. Copeptin improved age/NIHSS model’s discrimination, copeptin, MR-proANP, and NT-proBNP improved its calibration and overall performance. In the validation dataset (450 patients, median age 73 years, quartiles 66–81; 54% men; median NIHSS score 8, quartiles 3–14), copeptin was independently associated with various definitions of poor outcome and also mortality. Copeptin did not increase model’s discrimination but it did improve calibration and overall model performance.

**Discussion:**

Copeptin, NT-proBNP, and MR-proANP improved modest but consistently the predictive performance of established prognostic scores in patients with mild AIS. Copeptin was most consistently associated with poor outcome in patients with moderate to severe AIS, although its added prognostic value was less obvious.

## Graphical abstract

**Graphical abstract:**
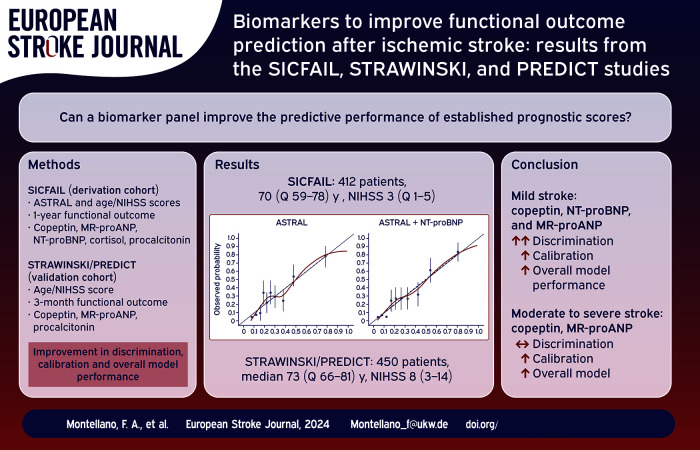

## Introduction

An accurate prediction of functional outcome is essential in acute ischemic stroke (AIS) management in order to inform patients and their relatives and tailor therapeutic, preventive, and rehabilitation measures accordingly.^1^ However, even clinicians with stroke expertise may poorly predict clinical outcomes of individual patients.^2,3^ To support clinicians in predicting functional outcome, numerous prognostic scores have been developed in recent decades.^4^ Nevertheless, existing scores might be not accurate enough to allow reliable clinical decision-making due to limited predictive properties.^5^ It has been proposed, for example, by the PROGnosis RESearch Strategy (PROGRESS) partnership, that the addition of novel prognostic factors may be an alternative to update or improve the performance of existing prognostic scores.^6^ Blood-based biomarkers might provide such additional prognostic information, that is not currently available in clinical routine.^7^ Blood-based biomarkers have been investigated extensively and a recent systematic review reported the consistent association of several biomarkers with poor outcome.^7^ However, the few studies that have examined their added prognostic value on top of existing scores^7^ have focused mostly on the improvement of discrimination (i.e., the ability of a model to assign higher risks to patients experiencing the outcome), overlooking other relevant performance measures of prognostic scores such as calibration (i.e., agreement between observed outcomes and predictions). Among existing scores, the Acute Stroke Registry and Analysis of Lausanne (ASTRAL) score^8^ is currently considered the best performing model according to a systematic review^4^ and head-to-head comparison.^5^ However, a simple model based on age and initial stroke severity (National Institutes of Health Stroke Scale [NIHSS])^9^ is the hitherto most used alternative in studies reporting on the incremental value of biomarkers beyond clinical variables.^7^

Therefore, we investigated whether a panel of blood-based biomarkers of three different domains (cardiac, inflammation, stress) selected via a recently updated systematic review^7^ may improve the predictive performance in terms of discrimination, calibration, and overall performance over best practice prognostic models exemplified by the ASTRAL score and the age/NIHSS model.

## Methods

### Data sources

The Stroke Induced Cardiac FAILure in mice and men (SICFAIL) cohort study intended to describe the natural course of cardiac function after AIS (clinical trial registration: DRKS00011615).^10^ Patients ⩾18 years with symptoms suggestive of AIS (World Health Organization definition^11^) were recruited at the Stroke Unit, Department of Neurology, University Hospital Würzburg, Germany. We used this cohort as derivation dataset.

The multicentric PREDICT cohort intended to identify predictors of post-stroke pneumonia and recruited patients ⩾18 years with AIS in any territory and within 36 h of symptom onset with NIHSS ⩾ 1 (clinical trial registration: NCT01079728).^12^

The STRoke Adverse outcome is associated WIth NoSoKomial Infections (STRAWINSKI) multicentric, randomized clinical trial investigated whether a procalcitonin-guided antibiotic therapy could improve the functional outcome of patients ⩾18 years with moderate to severe (NIHSS > 9 points) AIS in the middle cerebral artery territory (clinical trial registration: NCT01264549).^13^ Exclusion criteria and baseline investigations of all studies are listed in the supplemental material. We used a pooled dataset of the STRAWINSKI and PREDICT studies for external validation.^14^

### Baseline investigation

Demographic characteristics, comorbidities, pre-stroke functional status, and lifestyle factors were documented at baseline in all studies (for definitions see supplemental material). Patients with symptoms suggestive of acute AIS underwent routine diagnostic and etiological workup at the respective centers (see supplemental material).^10,12,13^

### Blood-based biomarkers

In SICFAIL, fasting blood samples were collected the morning after enrollment, at a median of 3 (quartiles 2–4) days after symptom onset. Blood samples in the STRAWINSKI and PREDICT studies were collected at a median of 1 day (quartiles 1–1) after symptom onset. Additional samples were drawn at days 2–4.

Based on a systematic review of the literature,^7^ we selected five biomarkers from three domains with previous evidence showing an incremental value over established prognostic markers that could be measured with kits available in routine clinical care: stress domain (copeptin; cortisol), inflammatory domain (procalcitonin), and cardiac domain (N-terminal pro-B-type natriuretic peptide, NT-proBNP; mid-regional pro-atrial natriuretic peptide, MR-proANP). For external validation, one cardiac (MR-proANP), stress (copeptin), and inflammatory (procalcitonin) biomarker were available for modeling. For details regarding sample storage and biomarker measurement see the supplemental material.

### Endpoints and follow-up

In SICFAIL, we defined poor outcome as major disability or death (mRS > 2) 1 year after stroke. In STRAWINSKI/PREDICT, poor outcome was defined as mRS > 2 three months after AIS, with further sensitivity analyses including mRS 4-6, mRS 5-6, and death. Outcome assessment was performed in all studies blinded to biomarker data.

### Standard protocol approvals, registrations, and patient consents

All studies complied with the Declaration of Helsinki and were approved by the respective Ethics Committee (SICFAIL: 176/13; PREDICT: EA1/216/09, PR_IR_170-2012; STRAWINSKI: EA1/267/10, AS 30(a)/2011, 2013-0195, TFS-ANT-2012-01; for details see supplemental material). All patients or their legal representatives provided written informed consent.

### Statistical analysis

We report standard descriptive summary statistics for the complete cohorts and groups with poor and good outcome. In line with current statistical guidance,^15^ we do not report significance tests for the comparison between groups in the descriptive tables. We used multivariable logistic regression to identify biomarkers independently associated with poor outcome after adjustment for the ASTRAL or age/NIHSS scores and report odds ratios (OR) with 95% confidence intervals (CI). In a sensitivity analysis we further adjusted for atrial fibrillation. Furthermore, we investigated the association of the biomarkers with varying definitions of poor outcome and in patients with moderate to severe stroke (NIHSS > 5). Due to limited statistical power in SICFAIL, these sensitivity analyses were done only in the STRAWINSKI/PREDICT dataset. Concentrations of biomarkers were logarithmically transformed due to skewed distribution. Reported ORs correspond to logarithmic increments of base 10.

We evaluated the performance of the ASTRAL score in the SICFAIL dataset.^8 [Bibr bibr9-23969873241250272]^ First, we calculated the sum score for each patient using the tables provided in the development paper.^8^ Then, we calculated a logistic regression including the ASTRAL score as the only predictor, allowing both coefficient and intercept to be freely estimated (logistic recalibration). We derived a second model including only age and NIHSS,^9 [Bibr bibr9-23969873241250272]^ since most available studies reported the incremental value of blood-based biomarkers over a model consisting of these two variables.^7^ However, as the original model was developed to predict a different outcome (Barthel Index ⩾ 95),^9^ we allowed for free estimation of coefficients and intercept. We externally evaluated the age/NIHSS model in the pooled dataset of STRAWINSKI/PREDICT, as previously described by Vergouwe et al.:^16^ (i) using the linear predictor of the original model with its coefficient set to 1 and intercept to 0, (ii) allowing the intercept to be freely estimated (recalibration in the large), (iii) allowing both the intercept and the coefficient of the linear predictor to be freely estimated (logistic recalibration), and (iv) allowing the coefficients for age and NIHSS and the intercept to be recalculated in the STRAWINSKI/PREDICT dataset (model revision). The respective coefficients and intercepts for each model are presented in the supplemental Table S8 (for details see supplemental material).^16,17^ We did not validate the ASTRAL score in the STRAWINSKI/PREDICT dataset because not all required variables were documented prospectively and their retrospective collection was not possible.

We investigated the improvement in predictive performance by generating a logistic regression including the ASTRAL or age/NIHSS models and selected biomarkers as independent variables. We assessed discrimination using the area under the receiver operator curve (AUROC). We used the DeLong test to investigate the increase in the AUROC provided by the addition of the different biomarkers. We assessed calibration with plots of predicted versus observed risk and report Harrell’s Emax (maximal absolute difference between the smoothed calibration curve and the diagonal line denoting best fit) and Eavg (average absolute difference in predicted and calibrated probabilities).^18^ We evaluated the overall model’s performance using the rescaled Brier score (range 0–1), with higher values indicating better performance.^19^ We provide a detailed explanation of these statistical measures in the supplemental material. We report bootstrapped 95% CI for the four performance metrics (Brier score, AUROC, Emax, and Eavg) based on 500 resampled replicates. We investigated proportion of the variance for functional outcome explained by single independent variables using Nagelkerke–Cox–Snell–Maddala–Magee R^2^ test. Statistical analyses were performed using R (version 4.1.2.).

## Results

### SICFAIL study

Between January 2014 and February 2017, 696 with patients with AIS were recruited.^10^ Blood samples were available from 544 of 696 patients (78%). Baseline characteristics did not differ between patients with and without available samples,^20^ while they differed only regarding glomerular filtration rate between patients who did and did not take part of the follow-up (supplemental Table S1). Calculation of the ASTRAL score was feasible for 520 patients (median 18 points, quartiles 16–21). Of those, 1-year follow-up was available from 412 (81%). Median age of included patients was 70 years (quartiles 59–78), median NIHSS score on admission was 3 points (quartiles 1–5) and 261 (63%) were men (Table 1).

**Table 1.** Characterization of the SICFAIL study population.

Variable	All patients (*n* = 412)	Good outcome (mRS ⩽ 2) (*n* = 290)	Poor outcome (mRS ⩾ 3) (*n* = 122)
Demographics			
Age (years)	70 (59–78)	66 (57–76)	76.5 (70–83)
Male sex	261 (63)	197 (68)	64 (52)
NIHSS	3 (1–5)	2 (1–4)	4 (2–9)
ASTRAL score	18 (16–21)	17 (15–20)	21 (18–26)
Etiology			
Large artery atherosclerosis	55 (13)	39 (13)	16 (13)
Cardioembolism	121 (30)	71 (25)	50 (41)
Small artery occlusion	56 (14)	45 (16)	11 (9)
Other cause	14 (3)	10 (3)	4 (3)
Undetermined	163 (40)	125 (43)	41 (34)
Risk factors			
Atrial fibrillation	98 (24)	47 (16)	51 (42)
Hypertension	308 (75)	202 (70)	106 (87)
Diabetes mellitus	111 (27)	65 (23)	46 (39)
Previous stroke	43 (10)	25 (9)	18 (15)
Hyperlipidemia	114 (28)	81 (28)	33 (28)
Coronary heart disease	74 (18)	43 (15)	31 (25)
Heart failure	29 (7)	14 (5)	16 (13)
Estimated glomerular filtration rate (mL/min/1.73 m^2^)	87 (71–96)	88 (73–99)	81 (62–92)
Biomarkers			
NT-proBNP (pg/mL)	276 (89–940)	188 (65–606)	753 (303–1991)
MR-proANP (pmol/L)	122 (74–2017)	101 (69–171)	198 (119–292)
Procalcitonin (ng/mL)	0.05 (0.03–0.07)	0.04 (0.03–0.07)	0.05 (0.03–0.09)
Copeptin (pmol/L)	7.9 (5.3–13.6)	7.2 (5.0–7.3)	12 (6.4–23.9)
Cortisol (μg/dL)	21.0 (16.8–25.7)	20.4 (15.9–24.7)	22.7 (18.6–27.2)
Events during follow-up			
Any stroke	35 (8.5)	23 (7.9)	12 (9.8)
Myocardial infarction	1 (0.2)	1 (0.3)	0
Pneumonia requiring rehospitalization	3 (0.7)	1 (0.3)	2 (1.6)

mRS: modified Rankin scale; NIHSS: National Institutes of Health Stroke Scale; ASTRAL: Acute Stroke Registry and Analysis of Lausanne; NT-proBNP: N-terminal B-type natriuretic peptide; MR-proANP: mid-regional proatrial natriuretic peptide.

Data are median (quartiles) or n (percent).

### STRAWINSKI/PREDICT studies

Four-hundred and eighty-six AIS patients were recruited between January 2010 and December 2012 in the PREDICT study.^12^ Between February 2011 and April 2014, 227 patients with moderate to severe stroke were recruited in the STRAWINSKI study.^13^ Twenty-nine patients were recruited in both studies.^14^ Of a total 683 patients (from both studies), blood samples were available in 573 patients, and measurements for the three biomarkers MR-proANP, procalcitonin, and copeptin were available from 545. Three-month functional outcome was available in 450 patients of them. We restricted the external validation to these patients. The median age was 73 years (quartiles 66–81), median NIHSS score on admission was 8 (quartiles 3–14) points and 242 (54%) were men (Table 2).

**Table 2.** Characterization of the pooled STRAWINSKI and PREDICT population.

Variable	All patients (*n* = 450)	Good outcome (mRS ⩽ 2) (*n* = 195)	Poor outcome (mRS ⩾ 3) (*n* = 255)
Demographics			
Age (years)	73 (66–81)	69 (61–76)	78 (70–84)
Male sex	242 (54)	122 (63)	120 (47)
NIHSS	8 (3–14)	4 (2–6)	13 (8–17)
Etiology			
Large artery atherosclerosis	137 (31)	72 (37)	65 (26)
Cardioembolism	165 (37)	48 (24)	117 (46)
Small artery occlusion	40 (9)	28 (14)	12 (5)
Other determined cause	15 (3)	7 (4)	8 (3)
Undetermined etiology	91 (20)	40 (21)	51 (20)
Risk factors			
Atrial fibrillation	161 (36)	43 (23)	118 (46)
Hypertension	376 (84)	154 (79)	222 (87)
Diabetes mellitus	119 (27)	37 (19)	82 (32)
Hyperlipidemia	234 (52)	103 (53)	131 (52)
Previous stroke	90 (20)	34 (18)	56 (22)
Biomarkers			
MR-proANP (pmol/L)	156 (90–257)	114 (71–174)	206 (116–321)
Procalcitonin (μg/L)	0.034 (0.023–0.052)	0.030 (0.022–0.044)	0.038 (0.023–0.066)
Copeptin (pmol/L)	10.5 (6.1–26.1)	7.3 (5.0–13.7)	17.8 (7.8–35.8)

mRS: modified Rankin scale; NIHSS: National Institutes of Health Stroke Scale; MR-proANP: midregional proatrial natriuretic peptide.

Data are median (quartiles) or *n* (percent).

### ASTRAL score

**Figure 1.**
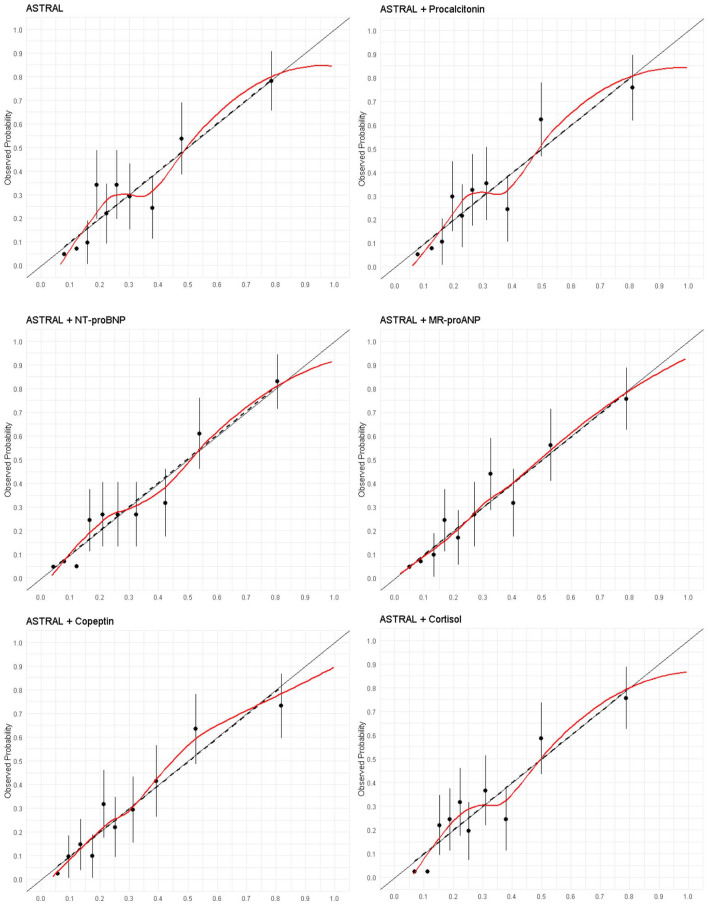
Calibration plots for the prediction of poor outcome using the ASTRAL score and single biomarkers in the SICFAIL dataset. Predicted probability in the X-axis.

Discrimination (AUROC) of the ASTRAL score in the SICFAIL dataset was good (0.76, 95% CI 0.71–0.81), while calibration (Figure 1) was acceptable (Emax0.08 (95% CI 0.04–0.18)).

### Age/NIHSS model

**Figure 2.**
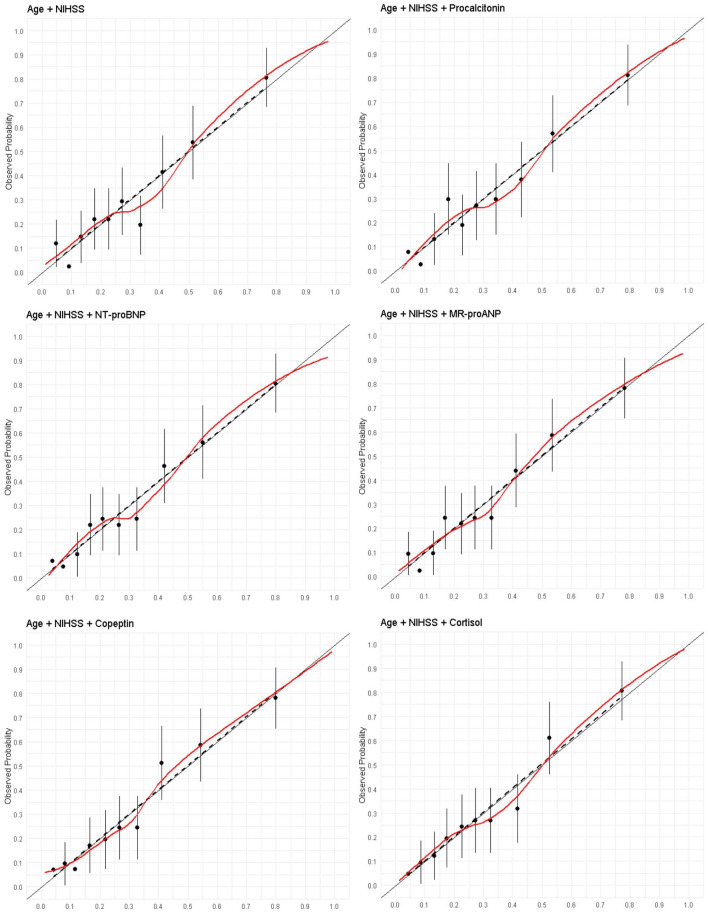
Calibration plots for the prediction of poor outcome using a model including age and the NIHSS score and single biomarkers in the SICFAIL dataset. Predicted probability in the X-axis.

**Figure 3.**
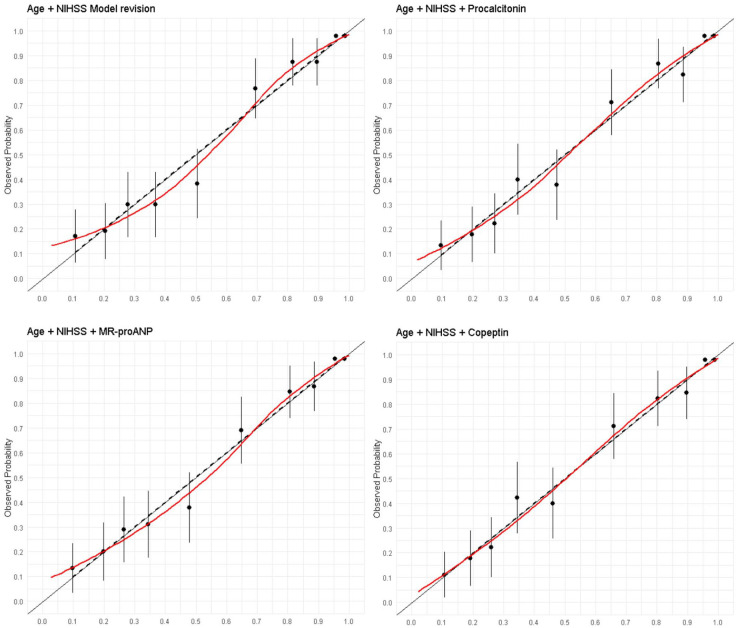
Calibration plots for the prediction of poor outcome using a model including age and the NIHSS score and single biomarkers in the STRAWINSKI/PREDICT dataset. Predicted probability in the X-axis.

In the SICFAIL derivation dataset the age/NIHSS model showed good discrimination with AUROC 0.77 (95% CI 0.73–0.82), and satisfactory calibration with Emax of 0.06 (95% CI 0.03–0.18; Figure 2). In the derivation dataset, stroke severity alone explained 14.3% of the observed variance. In the STRAWINSKI/PREDICT dataset, the age/NIHSS model showed very good discrimination (AUROC 0.86, 95% CI 0.83–0.90) and overall performance (Brier score 0.38, 95% CI 0.30–0.45), while calibration was poorer (Emax 0.10, 95% CI 0.06–0.17; Figure 3). Recalibration in the large, logistic calibration and model revision did not improve discrimination (Supplemental Table S10), although rescaled Brier score and calibration improved (Supplemental Table S7). In the validation dataset, stroke severity alone explained 44.9% of the observed variance. Coefficients and intercept are provided in supplemental Table S8.

### Blood-based biomarkers and prognostic performance of the ASTRAL score in the SICFAIL cohort

In the SICFAIL cohort, all biomarkers were associated at the univariable level with poor outcome (Table 3). Copeptin, NT-proBNP, MR-proANP, and cortisol were associated with poor outcome after adjustment for the ASTRAL score, while procalcitonin was not (Table 3). After further adjustment for atrial fibrillation, cortisol was no longer associated with poor outcome (supplemental Table S3). The addition of copeptin (AUROC 0.79, 95% CI 0.74–0.84), NT-proBNP (AUROC 0.80, 95% CI 0.76–0.84), and MR-proANP (AUROC 0.79, 95% CI 0.75–0.84) led to similar improvements in discrimination (all *p* < 0.05 when compared to ASTRAL score), calibration, and overall model’s performance. Cortisol and procalcitonin did not improve any performance measure of ASTRAL (supplemental Table S7). The best discrimination was observed with the combination of copeptin and one cardiac marker (AUROC 0.80, 95% CI 0.76–0.85 for MR-proANP; 0.81, 95% CI 0.77–0.85 for NT-proBNP), although this combination did not excel the models including solely ASTRAL and one cardiac marker (*p* = 0.11 for MR-proANP; *p* = 0.078 for NT-proBNP, supplemental Table S9). The models including copeptin and one cardiac marker yielded the highest Brier scores: 0.24 (95% CI 0.15–0.35 for MR-proANP, and 0.25 (95% CI 0.17–0.36) for NT-proBNP (supplemental Tables S7). Calibration plots are depicted in Figure 1.

**Table 3.** Association of selected biomarkers with poor outcome in univariable and multivariable logistic regression analysis in the SICFAIL cohort and in the STRAWINSKI/PREDICT pooled dataset.

Biomarker	Odds ratio* (95 % CI)	Odds ratio** (95 % CI)	Odds ratio*** (95 % CI)
Copeptin			
SICFAIL	5.69 (3.10–10.42)	2.93 (1.57–5.45)	3.29 (1.75–6.18)
STRAWINSKI/PREDICT	6.44 (3.87–10.70)	2.11 (1.11–3.98)	
Procalcitonin			
SICFAIL	2.07 (1.18–3.64)	1.17 (0.59–2.31)	1.22 (0.60–2.45)
STRAWINSKI/PREDICT	1.11 (0.97–1.27)	0.99 (0.85–1.16)	
Mid-regional pro atrial natriuretic peptide			
SICFAIL	15.49 (7.05–34.02)	3.74 (1.39–10.01)	6.01 (2.46–14.67)
STRAWINSKI/PREDICT	13.98 (7.05–27.74)	1.76 (0.69–4.48)	
N-Terminal B-type natriuretic peptide			
SICFAIL	3.72 (2.63–5.26)	2.15 (1.43–3.24)	2.52 (1.71–3.71)
Cortisol			
SICFAIL	16.60 (3.50–78.72)	4.43 (0.81–24.32)	5.74 (1.04–31.54)

Odds ratios are reported for logarithmic increases of base 10.

* Univariable analysis. **Adjusted for age and NIHSS. ***Adjusted for ASTRAL score.

### Blood-based biomarkers and prognostic performance of the age/NIHSS model in the SICFAIL cohort

Copeptin, NT-proBNP, MR-proANP, and cortisol were associated with poor outcome after adjustment for age and NIHSS, while procalcitonin was not (Table 3). Compared to age/NIHSS alone (AUROC 0.77, 95% CI 0.73–0.82), copeptin improved discrimination (0.80, 95% CI 0.76–0.84, *p* = 0.022), while the other biomarkers did not (see supplemental Tables S6 andS9). NT-proBNP, MR-proANP, and copeptin improved calibration (Figure 2), reducing Emax from 0.06 to 0.03–0.02 and improving the Brier score up to 0.24 for both copeptin and NT-proBNP (supplemental Table S7).

### Blood-based biomarkers and prognostic performance of the age/NIHSS model in the STRAWINSKI/PREDICT dataset

In the validation dataset, all biomarkers were associated with poor outcome at univariable level, but it remained significant only for copeptin after adjustment for age and NIHSS (Table 3). This association was stable across different mRS cutoffs (supplemental Table S5) and time points of sampling (days 1–4, supplemental Table S4), but not in the group of patients with NIHSS > 5, in which copeptin was only associated with very poor outcome (mRS 5–6) and death (supplemental Table S6). No studied biomarker improved discrimination significantly (*p* > 0.05 for all comparisons against age/NIHSS; supplemental Table S11). However, after model revision, the addition of MR-proANP, copeptin, or procalcitonin improved calibration. Copeptin led to the largest improvement in calibration (Emax reduction from 0.06 to 0.01; supplemental Table S7).

## Discussion

Our study showed that copeptin, MR-proANP, and NT-proBNP led to modest but similar and consistent improvements in discrimination, calibration, and overall performance of both the ASTRAL and age/NIHSS models for the prediction of poor 1-year outcome in patients with mostly mild to moderate AIS. Cardiac biomarkers improved calibration and overall performance of the ASTRAL and age/NIHSS models, while it improved discrimination of the ASTRAL score in the derivation dataset only. We observed no improvement in discrimination but modest improvements in calibration with the addition of some biomarkers in the pooled STRAWINSKI/PREDICT external validation dataset, including mostly patients with moderate to severe stroke and 3-month follow-up. Our work adds to the existing literature by demonstrating that blood-based biomarkers can also improve calibration and overall model’s performance, independently of improvement in discrimination for the prediction of poor outcome after AIS.

The integration of biomarker measurements in the clinical practice can be challenging and dichotomization into normal/abnormal categories is common. This approach, while intuitive, is both biologically implausible and statistically inefficient.^21^ In addition, we can speculate that the use of cutoffs may lead practitioners to make decisions based on individual biomarkers, although a single predictor is rarely a reliable estimator of prognosis.^22^ However, using continuous variables like biomarkers in their continuous scale (the methodologically recommended approach) may be difficult to interpret in clinical practice. Using blood-based biomarkers to update or to optimize existing clinical models^6^ allows harmonizing the need for clinical actionability with best analytical practice. Despite multiple calls to action, to date only few publications have implemented this approach in biomarker prognostic research after AIS. Of those, the majority investigated only the improvement in discrimination over a simple age/NIHSS model, neglecting the assessment of calibration and overall model performance as well as more complex prognostic models, despite current best practice recommendations.^23^ In a previous systematic review of prognostic stroke biomarkers, only 5% of identified studies investigated calibration. Of those, the majority reported only the Hosmer–Lemeshow test,^7^ which has limited statistical power and reduced informative value regarding the type and extent of miscalibration.^23^ Therefore, current statistical guidance suggests not assessing calibration using this test.^23^ Our work adds to the existing literature by demonstrating that blood-based biomarkers can also improve calibration (and overall model’s performance), independently of improvement in discrimination for the prediction of poor outcome after AIS. Calibration is especially important when supporting risk-based decision-making,^24^ since the systematic over- or underestimation of risk can render a prognostic model useless, despite good discrimination. In the case of functional outcome, strong over- or underestimation of the risk of poor outcome could lead to unwanted decisions regarding, for example, early withdrawal of life sustaining therapies in patients who otherwise may have had an acceptable outcome. Our work underscores the importance of integrating biomarkers into existing prognostic models as an alternative to successfully bridging the gap between biomarker research and implementation in routine clinical care, as opposed to considering them in isolation, for example, using cutoffs. Moving forward, further research is needed to investigate the clinical implications of more accurate risk prediction in routine care, which might require conducting cluster-randomized trials to properly assess the benefits (e.g., continuation of therapy in patients with predicted good outcome despite current clinical status suggesting otherwise) or potential risks (e.g., improving survival at the expense of increased disability) of routine implementation of prognostic scores. Importantly, the existence of accurate prognostic models is a prerequisite for the conduction of such studies.

The incremental value of copeptin is consistent with the previous published literature,^7^ although the pathophysiologic link between copeptin and poor outcome is unclear.^25^ Importantly, the association of copeptin with poor outcome has been identified in diseases other than stroke, like myocardial infarction,^26^ suggesting that the additional information provided (likely a marker of internal homeostasis), is not exclusive to stroke and beyond routinely collected clinical variables.^25^ Remarkably, especially in the validation cohort consisting of moderate to severe strokes, copeptin was independently associated with all possible definitions of disability based on mRS-cutoffs and with mortality and at all time points from days 1 to 4. Despite the lack of improvement in discrimination, copeptin improved calibration and overall model performance. Also the cardiac markers MR-proANP and NT-proBNP consistently improve model’s prognostic performance. In the derivation dataset we provide a head-to-head comparison between these markers, showing that both improve the model’s performance to a similar extent in all measures, probably because they depict the same pathophysiological mechanism. The improvement in performance may be mediated by etiology, since natriuretic peptides are associated with cardioembolism.^27^ However, after adjustment for atrial fibrillation both natriuretic peptides were still associated with poor outcome. Further, natriuretic peptides are associated with cardiac comorbidities (e.g., heart failure),^28^ which in turn is associated with poor outcome.^29^ Therefore, the improvement in prognostic performance may be mediated by a more accurate prediction of cardiac-related risk of poor outcome. Importantly, our works intends to optimize outcome prediction after AIS using biomarkers and our results should not be interpreted as causal effects.

We found no improvement in discrimination in the validation cohort. Two elements in our study suggest that this finding is explained by case mix. First, stroke severity explained only 14.3% of the variance regarding 1-year functional outcome in the derivation dataset (mostly mild strokes), compared to 44.9% of the variance explained at 3 months in patients with mostly moderate to severe stroke. The results are consistent with previous data (*n* = 614, median NIHSS = 5)^30^ showing that NIHSS accounts for 25% of the explained variance regarding functional outcome at 3 months. Second, among patients of the validation cohort with NIHSS > 5, copeptin was only associated with very poor outcome (mRS 5–6) and mortality but not with poor outcome defined as mRS 3–6. Importantly, the age/NIHSS model in the validation dataset (AUROC 0.86, 95% CI 0.83–0.90) outperformed that in the derivation dataset (AUROC 0.77, 95% CI 0.73–0.82), leaving little room for improvement with the addition of new variables. The relevance of initial stroke severity for adjustment is reinforced by the remarkable consistency in the magnitude of the odds ratios at the univariable level, despite the different time points of outcome assessment. However, the latter cannot be ruled out as a further possible source of discrepancy. Taken together, our data suggests that the relevance of stroke severity as a determinant of functional outcome depends on (1) stroke severity, being more important in more severely affected patients, and (2) its relevance may be higher shortly after the index event and diminish with time. Furthermore, it must be stressed that comparison of rank statistics (such as the AUROC) is a procedure that does not reward sufficiently extreme predictions, leading to reduced statistical power.^24^ Thus, assessment of prognostic relevance cannot be based solely on improvement of discrimination.

Importantly, our results do not permit to conclude that a simple age/NIHSS model outperforms the ASTRAL score, since the calculation of the later was performed using the tables provided in the development paper and the linear predictor could not be calculated, because the intercept was not reported. Thus, we cannot exclude that rounding error affected the overall performance of the ASTRAL score.

The major strength of this work is the structured approach we undertook to evaluate the integration of blood-based biomarkers for functional outcome prognostication, building up on our previous systematic review^7^ and using prospectively collected (and in the case of the STRAWINSKI/PREDICT dataset multicentric) data.^10,12–14^ Importantly, we evaluated the incremental prognostic value not only regarding discrimination, but also calibration and overall model’s performance. Furthermore, by reanalyzing data from the STRAWINSKI/PREDICT dataset in the external validation,^14^ we were able to gain relevant insights regarding the (possibly) distinctive role of biomarkers in poor outcome prediction in patients with different stroke severities and at different time points after AIS, which would not have been possible otherwise. Our study has, however, limitations. First, we performed a complete-case analysis. While the follow-up rate was around 80%, in line with comparable previous studies,^31^ and although only estimated glomerular filtration rate differed between patients participating in follow-up from those lost-to-follow-up, we cannot completely exclude that lost-to-follow-up mechanisms other than missing-completely-at-random may have introduced bias in our results. However, the lack of differences between groups in terms of age, stroke severity on admission, ASTRAL score, etiology, comorbidities, or biomarker levels are reassuring. Second, due to restrictions of the local ethics committee, patients were recruited in SICFAIL only if they were able to provide informed consent themselves or a legal representative was available, which determined the recruitment of mostly mild strokes. While the differing stroke severity profiles between SICFAIL and STRAWINSKI/PREDICT allowed us to gain some relevant insights, as stated above, they also represent a limitation regarding the exact replication of the results of the derivation cohort. The limitations of the ethics committee also resulted in a later time point of blood sampling in the derivation cohort. However, the remarkable consistency in the magnitude and direction of the ORs suggests that this limitation has not substantially influenced our results. Further, additional measurements in the validation cohort of copeptin, MR-proANP, and procalcitonin at days 2-4 did not significantly change the results, in line with previous research showing that MR-proANP’s predictive value remains unaltered during the first days after the index event, despite modest variations in serum concentration.^32^ Third, because the PREDICT/STRAWINSKI studies were not conceived as a validation cohort of SICFAIL, the time points of outcome assessment also differed between datasets. Caution is advised when directly comparing the performance between outcomes in the derivation and validation dataset, since functional outcome can continue improving beyond 3 months after AIS, especially in patients with severe stroke.^33^ Fourth, because not all variables required to calculate the ASTRAL score were available in the STRAWINSKI/PREDICT studies, the investigation of this score was not possible in the validation dataset. Fifth, we were not able to perform sensitivity analyses in the derivation dataset due to the low number of patients with severe stroke, resulting in limited statistical power. Sixth, although not all five biomarkers investigated in the derivation dataset were available in the validation dataset, we investigated at least one biomarker of each domain (cardiac, inflammation, stress). Lastly, the panel of biomarkers was selected based on a previous systematic review and we cannot exclude that other promising markers can similarly improve model performance.

In summary, our results suggest that: (1) copeptin, NT-proBNP, and MR-proANP can improve discrimination, calibration, and overall performance of existing models to predict long-term poor outcome in patients with mild to moderate AIS, and (2) the incremental value of blood-based biomarkers in the prediction of mid-term poor outcome in patients with moderate to severe AIS is unclear and needs to be addressed in further studies. The integration of blood-based biomarkers into existing models might ease the adoption of biomarkers in clinical routine.

## Supplemental Material

sj-doc-2-eso-10.1177_23969873241250272 – Supplemental material for Biomarkers to improve functional outcome prediction after ischemic stroke: Results from the SICFAIL, STRAWINSKI, and PREDICT studies Supplemental material, sj-doc-2-eso-10.1177_23969873241250272 for Biomarkers to improve functional outcome prediction after ischemic stroke: Results from the SICFAIL, STRAWINSKI, and PREDICT studies by Felipe A Montellano, Viktoria Rücker, Kathrin Ungethüm, Anna Penalba, Benjamin Hotter, Marina Giralt, Silke Wiedmann, Daniel Mackenrodt, Caroline Morbach, Stefan Frantz, Stefan Störk, William N Whiteley, Christoph Kleinschnitz, Andreas Meisel, Joan Montaner, Karl Georg Haeusler and Peter U Heuschmann in European Stroke Journal

sj-docx-1-eso-10.1177_23969873241250272 – Supplemental material for Biomarkers to improve functional outcome prediction after ischemic stroke: Results from the SICFAIL, STRAWINSKI, and PREDICT studies Supplemental material, sj-docx-1-eso-10.1177_23969873241250272 for Biomarkers to improve functional outcome prediction after ischemic stroke: Results from the SICFAIL, STRAWINSKI, and PREDICT studies by Felipe A Montellano, Viktoria Rücker, Kathrin Ungethüm, Anna Penalba, Benjamin Hotter, Marina Giralt, Silke Wiedmann, Daniel Mackenrodt, Caroline Morbach, Stefan Frantz, Stefan Störk, William N Whiteley, Christoph Kleinschnitz, Andreas Meisel, Joan Montaner, Karl Georg Haeusler and Peter U Heuschmann in European Stroke Journal
