# Supplementary material for: Biomarkers to improve functional outcome prediction after ischemic stroke: Results from the SICFAIL, STRAWINSKI, and PREDICT studies
Source: Eur Stroke J. 2024 May 6;9(4):968–80. doi: 10.1177/23969873241250272 (PMC11569564; doi:10.1177/23969873241250272)
Supplement: sj-docx-1-eso-10.1177_23969873241250272 – Supplemental material for Biomarkers to improve functional outcome prediction after ischemic stroke: Results from the SICFAIL, STRAWINSKI, and PREDICT studies [file sj-docx-1-eso-10.1177_23969873241250272.docx]

**Supplemental Material**

**Supplemental Methods**
Measures of prognostic performance
Standard Protocol Approvals, Registrations, and Patient Consents
Diagnostic Workup
Baseline investigation: Definitions
Blood-based biomarkers

**Table S1** Characterization of patients (non-responder analysis) participating and not participating of follow-up (lost-to-follow-up)
**Table S2** Association of selected biomarkers with poor outcome in univariable and multivariable logistic regression analysis in the SICFAIL cohort and in the STRAWINSKI/PREDICT pooled dataset (natural logarithmic transformation)
**Table S3** Association of selected biomarkers with poor outcome in multivariable logistic regression analysis in the SICFAIL cohort and in the STRAWINSKI/PREDICT pooled dataset after additional adjustment for atrial fibrillation.
**Table S4**. Association of selected biomarkers measured on days 2-4 with poor outcome in multivariable logistic regression analysis in the STRAWINSKI/PREDICT pooled dataset
**Table S5** Association of selected biomarkers with further definitions of poor outcome in multivariable logistic regression analysis in the STRAWINSKI/PREDICT pooled dataset
**Table S6.** Association of selected biomarkers in patients with moderate to severe stroke (NIHSS > 5) with different definitions of poor outcome in multivariable logistic regression analysis in the STRAWINSKI/PREDICT pooled dataset
**Table S7.** Model performance **Table S8**. Coefficients and intercept for different models including age and NIHSS
**Table S9:** Comparison between AUROCs including the ASTRAL score and additional biomarkers using the DeLong test in the SICFAIL dataset
**Table S10:** Comparison between AUROCs including age, stroke severity and additional biomarkers using the DeLong test in the SICFAIL dataset
**Table S11:** Comparison between AUROCs including age, stroke severity and additional biomarkers using the DeLong test in the STRAWINSKI/PREDICT dataset

**Supplemental Methods**

**Measures of prognostic performance**

“Discrimination, calibration, and overall performance are standard measures of performance of prognostic models.(1) **Discrimination**, the most reported of those, refers to the ability of a variable or model/score to assign higher values to those patients experiencing an outcome than to those not experience the outcome. This is done be establishing a rank statistic and comparing pairs of patients, in the case of our study, among patients with poor and good outcome. Taking the NIHSS as an example, patients with higher NIHSS scores tend to have poorer outcomes than those with lower NIHSS scores. All these cases **increase** the area under the receiver operator curve (AUROC), signifying a better predictive performance of the variable. However, a patient with a NIHSS of 2 points (caused by dense aphasia) may have a poorer outcome than a patient with an NIHSS of 3 points caused by mild sensory deficits, mild dysarthria and mild facial paralysis. In that case, this pairing will **reduce** the AUROC. Noticeably, although it is by far the most reported measure of model performance, the clinical value of the AUROC is not self-evident. Contrary to popular belief, the AUROC does not represent the percentage of patients correctly classified by the model.(1)

In contrast, **calibration**, an often-neglected measure, has a much direct correlate in clinical practice. Calibration reflects the **agreement** between the **predicted** and **observed probability** in the dataset. Suppose that the age/NIHSS score assigns a risk of 60% to a patient of having a mRS of 3-6 at one year after stroke. This is the **predicted probability**. In practical terms, this means that 6 out of 10 patients with this combination of age and NIHSS will experience a poor outcome. The **observed probability** is, in turn, the actual frequency in which a poor outcome is observed among those patients with that given combination of age and NIHSS. A perfect calibration would mean that indeed, for all possible combinations of age and NIHSS, the predicted probability always accurately matches the observed probability. In the example outlined above, risk underestimation would mean that instead of the predicted 60% risk of poor outcome, the observed probability would be e.g. 90%. Conversely, an observed probability of 40% would imply risk overestimation. Importantly, consequences of this risk under- or overestimation in clinical practice depend on patient’s or their relative’s preferences and have been insufficiently investigated. However, it is likely that severe risk overestimation could lead e.g. to withdrawal of life sustaining therapies in patients who otherwise could have had a better outcome.

The **scaled Brier score** used in our publication does not have a direct clinical interpretation, but it summarizes in one measure both^2^ discrimination and calibration.”

**Exclusion criteria**

Patients participating in an acute interventional study or with a discharge diagnosis other than AIS were excluded from the SICFAIL study. Patients with intracerebral hemorrhage, preexisting dysphagia, mechanical ventilation, antibiotic treatment or immunosuppressive therapy within 4 weeks or signs of infections at screening were excluded from the PREDICT study. Patients with lacunar infarction, suspected life expectancy <3 months (irrespective of the underlying cause), modified Rankin Scale (mRS) before AIS onset ≥4, pregnant/lactating, or those participating in other interventional trial were excluded from the STRAWINSKI study.

**Standard Protocol Approvals, Registrations, and Patient Consents**

The SICFAIL study was approved by the Ethics Committee of the Medical Faculty of the University of Würzburg (176/13). The PREDICT study was approved by the Ethics Committees of the Charité – Universitätsmedizin Berlin (EA1/216/09) for study centers located in Berlin and Hospital Vall d’Hebron Barcelona (PR_IR_170-2012) for the study center in Barcelona. The STRAWINSKI study was approved by the Ethics Committees of the Charité – Universitätsmedizin Berlin (EA1/267/10) for study centers located in Berlin, the ethics committee of the State Medical Association of Brandenburg (AS 30(a)/2011) for the study center located in Brandenburg, the Kantonale Ethikkommission Zürich (2013-0195) for the study center in Zürich, and the Hospital Vall d’Hebron Clinical Research Ethics Committee (TFS-ANT-2012-01) for the study center in Barcelona.

**Diagnostic Workup**

All patients underwent neuroimaging (computed tomography or magnetic resonance imaging), vascular imaging (Doppler, CT and/or MRI angiography), 12-lead electrocardiogram on admission, and ECG-monitoring at the stroke unit or intensive care unit. The etiological classification was done following the original Trial of Org 10172 in Acute Stroke Treatment (TOAST).(2) Therefore, patients with concurrent etiologies (e.g. atrial fibrillation and large-artery atherosclerosis or atrial fibrillation and small vessel disease) were classified as Undefined (UND).

**Baseline investigation: Definitions**

Within SICFAIL, hypertension was defined as self-reported history of hypertension or intake of antihypertensive medication. Atrial fibrillation was defined according to the medical records of the index event or a new diagnosis of atrial fibrillation during the index stay. Heart failure was defined according to the European Society of Cardiology guidelines(3), as previously described.(4) Glomerular filtration rate was estimated using the Chronic Kidney Disease Epidemiology Collaboration formula.(5)

**Blood-based biomarkers**

After centrifugation, serum and plasma samples of the SICFAIL study were stored at -80 °Celsius in the Interdisciplinary Bank of Biomaterials and Data Würzburg according to guidelines of the Organization for Economic Cooperation and Development.(6) Blood samples of the PREDICT and STRAWINSKI studies were stored -80 °Celsius in the NeuroHub biobank of the NeuroCure Clinical Research Center, Charité Universitätsmedizin Berlin.

The following assays were used to determine the levels of the identified blood-based biomarkers in the SICFAIL study: (i) NT-proBNP: Elecsys proBNP II (Roche Diagnostics, Mannheim, Germany; range 5–35000 pg/mL); (ii) MR-proANP (BRAHMS GmbH/Thermo Fisher Scientific, Hennigsdorf, Germany; range 2.1–1000 pmol/L); (iii) copeptin (BRAHMS GmbH/Thermo Fisher Scientific, Hennigsdorf, Germany; range: 0.7–500 pmol/L); (iv) procalcitonin (Atellica IM BRAHMS Procalcitonin, Siemens, Erlangen, Germany; range: 0.03–50.00 ng/mL); (v) cortisol (Atellica IM Cortisol, Siemens, Erlangen, Germany; range: 0.50–75.00 μg/dl). All measurements took place at a single time point at the end of the study by technicians blinded to the clinical outcome of patients. NT-proBNP measurements were performed at a single time point after study completion at the local laboratory facilities of the University Hospital Würzburg. The remaining measurements took place at a single time point after study completion at the Neurovascular research laboratory, Vall d’Hebron Institut de Recerca, Barcelona, Spain.

In the STRAWINSKI(7) and PREDICT studies,(8) the measurements of MR-proANP, copeptin, and ultrasensitive procalcitonin were performed using immunoassay BRAHMS KRYPTOR GmbH/Thermo Fisher Scientific, Hennigsdorf, Germany by technicians blinded to the clinical outcome of patients. MR-proANP and copeptin were measured centrally at a single time point after study completion at the Neurovascular research laboratory, Vall d’Hebron Institut de Recerca, Barcelona, Spain. Procalcitonin measurements took place either centrally (PREDICT) at the end of the study or locally (STRAWINSKI) upon patient recruitment.

**Table S1.** Characterization of patients (non-responder analysis) participating and not participating of follow-up (lost-to-follow-up)

|  | | | **Patients with available follow-up**  **(n=561)** | **Patients lost-to-follow-up**  **(n=135)** | **p-value** |
| --- | --- | --- | --- | --- | --- |
| **Demographics** |  | | |  |  |
| Age (y), | 71 (60.5-79) | | | 71 (58-78) | 0.54 |
| Male sex | 209 (63) | | | 77 (57) | 0.22 |
| NIHSS | 3 (1-5) | | | 3 (1-5) | 0.24 |
| ASTRAL score | 18 (16-21) | | | 19 (16-22) | 0.82 |
| **Etiology** |  | | |  | 0.92 |
| Large artery  atherosclerosis | 70 (12) | | | 14 (10) |  |
| Cardioembolism | 166 (30) | | | 40 (30) |  |
| Small artery occlusion, | 76 (14) | | | 19 (14) |  |
| Other cause, | | 18 (3) | | 3 (2) |  |
| Undetermined, | | 231 (41) | | 59 (44) |  |
| **Risk factors** | |  | |  |  |
| Atrial fibrillation | | 135 (24) | | 29 (21) | 0.53 |
| Hypertension | | 380 (68) | | 95 (70) | 0.55 |
| Diabetes mellitus | | 159 (29) | | 44 (33) | 0.37 |
| Previous stroke | | 65 (12) | | 17 (13) | 0.51 |
| Hyperlipidemia | | 151 (28) | | 38 (30) | 0.56 |
| Coronary heart disease | | 100 (18) | | 13 (10) | 0.32 |
| Estimated glomerular filtration rate (mL/min/1.73 m^2^) | | 87 (72-96) | | 89 (81-96) | 0.03 |
| **Biomarkers** | |  | |  |  |
| NT-proBNP (pg/mL) | | 272 (94-971) | | 291 (104-805) | 0.41 |
| MR-proANP (pmol/L) | | 122 (73-209) | | 115 (79-193) | 0.42 |
| Procalcitonin (ng/mL) | | 0.05 (0.03-0.07) | | 0.05 (0.03-0.07) | 0.53 |
| Copeptin (pmol/L) | | 8 (5.3-13.8) | | 8 (5.9-14.3) | 0.50 |
| Cortisol (μg/dL) | | 21 (16.9-27.5) | | 20 (16.7-23.8) | 0.26 |

Data are median (quartiles) or n (percent). NIHSS: National Institutes of Health Stroke Scale; ASTRAL: Acute Stroke Registry and Analysis of Lausanne; NT-proBNP: N-terminal B-type natriuretic peptide; MR-proANP: mid-regional proatrial natriuretic peptide

**Table S2.** Association of selected biomarkers with poor outcome in univariable and multivariable logistic regression analysis in the SICFAIL cohort and in the STRAWINSKI/PREDICT pooled dataset (natural logarithmic transformation)

|  | **Odds ratio* (95 % CI)** | **Odds ratio** (95 % CI)** | **Odds ratio*** (95 % CI)** |
| --- | --- | --- | --- |
| Copeptin  *SICFAIL*  *STRAWINSKI/PREDICT* | 2.13 (1.64-2.77)  2.22 (1.77-2.78) | 1.60 (1.21-2.10)  1.40 (1.05-1.85) | 1.68 (1.28-2.21)  - |
| Procalcitonin  *SICFAIL*  *STRAWINSKI/PREDICT* | 1.37 (1.08-1.75)  1.45 (1.19-1.77) | 1.07 (0.80-1.44)  1.24 (0.96-1.61) | 1.01 (0.80-1.48)  - |
| Mid-regional pro atrial natriuretic peptide  *SICFAIL*  *STRAWINSKI/PREDICT* | 3.29 (2.34-4.63)  3.25 (2.38-4.43) | 1.77 (1.15-2.73)  1.30 (0.86-1.97) | 2.18 (1.48-3.21)  - |
| N-Terminal B-type natriuretic peptide  *SICFAIL* | 1.77 (1.52-2.06) | 1.40 (1.17-1.67) | 1.49 (1.26-1.77) |
| Cortisol  *SICFAIL* | 3.39 (1.72-6.66) | 1.91 (0.91-4.00) | 2.14 (1.02-4.48) |

Odd ratios correspond to per-unit change of the log-transformed data. * Univariable analysis; **adjusted for age and NIHSS, ***adjusted for ASTRAL score

**Table S3.** Association of selected biomarkers with poor outcome in multivariable logistic regression analysis in the SICFAIL cohort and in the STRAWINSKI/PREDICT pooled dataset after additional adjustment for atrial fibrillation

|  | **Odds ratio* (95 % CI)** | **Odds ratio** (95 % CI)** |
| --- | --- | --- |
| Copeptin  *SICFAIL*  *STRAWINSKI/PREDICT* | 2.82 (1.51-5.26)  2.10 (1.11-3.99) | 2.93 (1.56-5.49) |
| Procalcitonin  *SICFAIL*  *STRAWINSKI/PREDICT* | 1.122 (0.57-2.24)  0.99 (0.85-1.16) | 1.11 (0.54-2.26) |
| Mid-regional pro atrial natriuretic peptide  *SICFAIL*  *STRAWINSKI/PREDICT* | 3.50 (1.21-10.07)  1.86 (0.77-4.97) | 5.34 (1.98-14.52) |
| N-Terminal B-type natriuretic peptide  *SICFAIL* | 2.16 (1.39-3.35) | 2.49 (1.62-3.83) |
| Cortisol  *SICFAIL* | 4.08 (0.75-22.28) | 4.78 (0.87-26.1) |

Odds ratios are reported for logarithmic increases of base 10. *adjusted for age and NIHSS and atrial fibrillation **adjusted for ASTRAL score and atrial fibrillation

**Table S4.** Association of selected biomarkers measured on days 2-4 with poor outcome in multivariable logistic regression analysis in the STRAWINSKI/PREDICT pooled dataset

|  | **Odds ratio* (95 % CI)** | **Odds ratio** (95 % CI)** |
| --- | --- | --- |
| Copeptin  *Day 2*  *Day 3*  *Day 4* | 6.79 (3.85-12.00)  5.79 (3.38-9.93)  6.14 (3.48-10.84) | 2.68 (1.32-5.42)  2.45 (1.22-4.95)  3.03 (1.50-6.10 |
| Procalcitonin  *Day 2*  *Day 3*  *Day 4* | 1.14 (0.99-1.31)  1.28 (1.01-1.61)  1.17 (0.99-1.38) | 0.96 (0.82-1.12)  1.02 (0.84-1.24)  1.03 (0.87-1.23) |
| Mid-regional pro atrial natriuretic peptide  *Day 2*  *Day 3*  *Day 4* | 12.75 (6.41-25.36)  9.90 (5.17-18.98)  9.72 (4.88-19.35) | 1.57 (0.60-4.15)  1.25 (0.50-3.11)  1.89 (0.82-4.42 |

Odds ratios are reported for logarithmic increases of base 10. *univariable analysis. **adjusted for age and NIHSS.

**Table S5.** Association of selected biomarkers with further definitions of poor outcome in multivariable logistic regression analysis in the STRAWINSKI/PREDICT pooled dataset

|  | **Odds ratio* (95 % CI) mRS 4-6** | **Odds ratio* (95 % CI) mRS 5-6** | **Odds ratio* (95 % CI) Mortality** |
| --- | --- | --- | --- |
| Copeptin  *STRAWINSKI/PREDICT* | l.95 (1.08-3.53) | 2.13 (1.09-4.18) | 2.29 (1.11-4.71) |
| Procalcitonin  *STRAWINSKI/PREDICT* | 0.93 (0.79-1.10) | 0.88 (0.72-1.08) | 0.88 (0.71-1.09) |
| Mid-regional pro atrial natriuretic peptide  *STRAWINSKI/PREDICT* | 1.71 (0.67-4.36) | 2.27 (0.67-7.67) | 4.79 (1.23-18.71) |

Odds ratios are reported for logarithmic increases of base 10. *adjusted for age and NIHSS. mRS: modified Rankin Scale.

**Table S6.** Association of selected biomarkers in patients with moderate to severe stroke (NIHSS > 5) with different definitions of poor outcome in multivariable logistic regression analysis in the STRAWINSKI/PREDICT pooled dataset

|  | **Odds ratio* (95 % CI) mRS 3-6** | **Odds ratio* (95 % CI) mRS 4-6** | **Odds ratio* (95 % CI) mRS 5-6** | **Odds ratio* (95 % CI) Mortality** |
| --- | --- | --- | --- | --- |
| Copeptin  *STRAWINSKI/PREDICT* | 1.35 (0.59-3.09) | 1.76 (0.89-3.44) | 2.43 (1.15-5.12) | 2.53 (1.14-5.63) |
| Procalcitonin  *STRAWINSKI/PREDICT* | 0.96 (0.78-1.19) | 0.95 (0.78-1.15) | 0.97 (0.75-1.25) | 1.03 (0.72-1.48) |
| Mid-regional pro atrial natriuretic peptide  *STRAWINSKI/PREDICT* | 1.74 (0.41-5.32) | 1.16 (0.39-3.47) | 2.88 (0.74-11.23) | 7.23 (1.51-34.68) |

Odds ratios are reported for logarithmic increases of base 10. *adjusted for age and NIHSS. mRS: modified Rankin Scale.

**Table S7: Model performance**

| Model | AUC (95% CI) | Brier Score | Emax | Eavg |
| --- | --- | --- | --- | --- |
| ASTRAL | | | | |
| ASTRAL | 0.76 (0.71-0.81) | 0.19 (0.10-0.31) | 0.08 (0.04-0.18) | 0.03 (0.01-0.05) |
| ASTRAL + NT-proBNP | 0.80 (0.76-0.84) | 0.24 (0.15-0.35) | 0.03 (0.02-0.13) | 0.01 (0.01-0.04) |
| ASTRAL + MR-proANP | 0.79 (0.74-0.84) | 0.22 (0.14-0.33) | 0.02 (0.02-0.14) | 0.01 (0.01-0.03) |
| ASTRAL + Copeptin | 0.79 (0.75-0.84) | 0.22 (0.14-0.34) | 0.10 (0.03-0.23) | 0.02 (0.01-0.05) |
| ASTRAL + Cortisol | 0.77 (0.72-0.82) | 0.19 (0.11-0.31) | 0.07 (0.04-0.19) | 0.02 (0.01-0.04) |
| ASTRAL + Procalcitonin | 0.76 (0.72-0.81) | 0.17 (0.09-0.30) | 0.07 (0.04-0.19) | 0.05 (0.01-0.05) |
| ASTRAL + Copeptin + NT-proBNP | 0.81 (0.77-0.85) | 0.25 (0.17-0.36) | 0.05 (0.02-0.17) | 0.01 (0.01-0.04) |
| ASTRAL + Copeptin + MR-proANP | 0.80 (0.76-0.85) | 0.24 (0.15-0.35) | 0.07 (0.02-0.20) | 0.02 (0.01-0.05) |
| ASTRAL + Copeptin + NT-proBNP + MR-proANP | 0.81 (0.77-0.86) | 0.26 (0.17-0.37) | 0.03 (0.02-0.15) | 0.01 (0.01-0.04) |
| Age/NIHSS | | | | |
| Age/NIHSS | 0.77 (0.73-0.82) | 0.22 (0.13-0.33) | 0.06 (0.03-0.18) | 0.02 (0.01-0.05) |
| Age/NIHSS +  NT-proBNP | 0.80 (0.76-0.84) | 0.24 (0.17-0.36) | 0.03 (0.02-0.12) | 0.01 (0.01-0.04) |
| Age/NIHSS+ MR-proANP | 0.79 (0.75-0.84) | 0.23 (0.15-0.34) | 0.03 (0.02-0.13) | 0.01 (0.01-0.04) |
| Age/NIHSS + Copeptin | 0.80 (0.76-0.84) | 0.24 (0.17-0.36) | 0.03 (0.02-0.13) | 0.02 (0.01-0.04) |
| Age/NIHSS + Cortisol | 0.78 (0.73-0.83) | 0.22 (0.14-0.33) | 0.05 (0.03-0.18) | 0.02 (0.01-0.04) |
| Age/NIHSS + Procalcitonin | 0.78 (0.73-0.83) | 0.22 (0.13-0.33) | 0.04 (0.02-0.18) | 0.02 (0.01-0.04) |
| Age/NIHSS + NT-proBNP + MR-proANP | 0.80 (0.76-0.84) | 0.25 (0.17-0.36) | 0.04 (0.02-0.14) | 0.02 (0.01-0.04) |
| Age/NIHSS + Copeptin + NT-proBNP | 0.81 (0.77-0.85) | 0.26 (0.17-0.37) | 0.04 (0.02-0.13) | 0.01 (0.01-0.04) |
| Age/NIHSS + Copeptin + MR-proANP | 0.80 (0.76-0.85) | 0.25 (0.17-0.36) | 0.04 (0.02-0.13) | 0.02 (0.01-0.04) |
| External validation | | | | |
| Age/NIHSS  (intercept=0, beta=1) | 0.86 (0.83-0.89) | 0.38 (0.30-0.44) | 0.1 (0.06-0.16) | 0.05 (0.03-0.09) |
| Age/NIHSS (recalibration in the large, intercept freely estimated, beta=1) | 0.86 (0.83-0.89) | 0.39 (0.32-0.45) | 0.04 (0.03-0.14) | 0.03 (0.01-0.06) |
| Age /NIHSS (logistic calibration, intercept and beta freely estimated) | 0.86 (0.83-0.89) | 0.39 (0.32-0.47) | 0.04 (0.01-0.11) | 0.01 (0-0.03) |
| Age/NIHSS  (model revision) | 0.87 (0.83-0.90) | 0.41 (0.22-0.49) | 0.06 (0.02-0.14) | 0.02 (0 – 0.04) |
| Age/NIHSS + MR-proANP | 0.87 (0.83-0.90) | 0.41 (0.33-0.49) | 0.04 (0.01-0.11) | 0.01 (0-0.03) |
| Age/NIHSS + Copeptin | 0.87 (0.84-0.90) | 0.41 (0.34-0.49) | 0.01 (0.01-0.09) | 0.004 (0-0.03) |
| Age/NIHSS + Procalcitonin | 0.87 (0.84-0.90) | 0.41 (0.34-0.49) | 0.02 (0.01-0.10) | 0.006 (0-0.03) |
| Age/NIHSS + MR-proANP + Procalcitonin | 0.87 (0.84-0.90) | 0.41 (0.34-0.49) | 0.02 (0.01-0.09) | 0.005 (0-0.03) |
| Age/NIHSS + MR-proANP + Copeptin | 0.87 (0.84-0.90) | 0.41 (0.34-0.49) | 0.01 (0.01-0.09) | 0.003 (0-0.03) |
| Age/NIHSS + Copeptin + Procalcitonin | 0.87 (0.84-0.91) | 0.42 (0.35-0.50) | 0.03 (0.01-0.09) | 0.01 (0-0.04) |

mRS: modified Rankin scale; NIHSS: National Institutes of Health Stroke Scale; ASTRAL: Acute Stroke Registry and Analysis of Lausanne; NT-proBNP: N-terminal B-type natriuretic peptide; MR-proANP: mid-regional proatrial natriuretic peptide

**Table S8: Coefficients and intercept for different models including age and NIHSS**

| Variable | Age + NIHSS  (SICFAIL, derivation) | Age + NIHSS  (STRANWISKI/  PREDICT)  Original model | Age + NIHSS  (STRANWISKI/  PREDICT)  Recalibration in the large | Age + NIHSS  (STRANWISKI/  PREDICT)  Logistic calibration | Age + NIHSS  (STRANWISKI/  PREDICT)  Model revision |
| --- | --- | --- | --- | --- | --- |
| Age | 0.06729 | - | - | - | 0.05883 |
| NIHSS | 0.17804 | - | - | - | 0.25402 |
| Linear predictor | - | 1 | 1 | 1.2130 | - |
| Intercept | -6.38000 | 0 | 0.3338 | 0.3923 | -5.99279 |

**Table S9: Comparison between AUROCs including the ASTRAL score and additional biomarkers using the DeLong test in the SICFAIL dataset**

|  | ASTRAL+  NT-proBNP | ASTRAL+  MR-proANP | ASTRAL +  Copeptin | ASTRAL +  Cortisol | ASTRAL +  Procalcitonin |
| --- | --- | --- | --- | --- | --- |
| ASTRAL | 0.0125 | 0.0246 | 0.0104 | 0.2507 | 0.7676 |
| ASTRAL+  NT-proBNP |  |  | 0.8287 |  |  |
| ASTRAL + MR-proANP |  |  | 0.7096 |  |  |
| ASTRAL+  NT-proBNP+  Copeptin | 0.0775 |  | 0.1248 |  |  |
| ASTRAL + MR-proANP+ Copeptin |  | 0.1102 | 0.268 |  |  |
| ASTRAL + MR-proANP+ NT-proBNP  +Copeptin | 0.1743 | 0.0961 | 0.2101 |  |  |

Reported values represent the respective p-value for the comparison

ASTRAL+MR-proANP + Copeptin vs. ASTRAL + NT-proBNP+Copeptin= p-value = 0.1677

**Table S10: Comparison between AUROCs including age, stroke severity and additional biomarkers using the DeLong test in the SICFAIL dataset**

|  | Age + NIHSS+ NT-proBNP | Age+NIHSS + MR-proANP | Age + NIHSS + Copeptin | Age + NIHSS + Cortisol | Age + NIHSS + Procalcitonin |
| --- | --- | --- | --- | --- | --- |
| Age/NIHSS | 0.06638 | 0.1153 | 0.02166 | 0.4234 | 0.69 |
| Age/NIHSS+  NT-proBNP |  |  | 0.8746 |  |  |
| Age + NIHSS + MR-proANP |  |  | 0.3227 |  |  |
| Age/NIHSS + NT-proBNP+  Copeptin | 0.06762 |  | 0.1903 |  |  |
| Age/NIHSS + MR-proANP+ Copeptin |  | 0.08653 | 0.4266 |  |  |
| Age/NIHSS + MR-proANP+ NT-proBNP  +Copeptin | 0.1314 | 0.07092 | 0.2858 |  |  |

Reported values represent the respective p-value for the comparison

Age/NIHSS vs Age + NIHSS + Cop + NT-proBNP: p= 0.007664

**Table S11: Comparison between AUROCs including age, stroke severity and additional biomarkers using the DeLong test in the STRAWINSKI/PREDICT dataset (external validation)**

|  | Age+NIHSS | Age+NIHSS + MR-proANP | Age + NIHSS + Copeptin | Age + NIHSS + Procalcitonin |
| --- | --- | --- | --- | --- |
| Age/NIHSS |  | 0.3917 | 0.1578 | 0.3288 |
| Age /NIHSS + MR-proANP |  |  |  |  |
| Age/NIHSS + MR-proANP+ Copeptin |  | 0.2457 | 0.8303 |  |
| Age/NIHSS + MR-proANP + PCT | 0.1527 | 0.3453 |  | 0.4639 |
| Age/NIHSS + MR-proANP+ Procalcitonin | 0.09274 |  |  |  |
| Age/NIHSS + MR-proANP+ Copeptin + Procalcitonin | 0.09333 |  |  |  |

Reported values represent the respective p-value for the comparison

References

1. Alba AC, Agoritsas T, Walsh M, Hanna S, Iorio A, Devereaux PJ, et al. Discrimination and Calibration of Clinical Prediction Models: Users’ Guides to the Medical Literature. JAMA. 2017 Oct 10;318(14):1377–84.

2. Adams HP, Bendixen BH, Kappelle LJ, Biller J, Love BB, Gordon DL, et al. Classification of subtype of acute ischemic stroke. Definitions for use in a multicenter clinical trial. TOAST. Trial of Org 10172 in Acute Stroke Treatment. Stroke. 1993 Jan;24(1):35–41.

3. Ponikowski P, Voors AA, Anker SD, Bueno H, Cleland JGF, Coats AJS, et al. 2016 ESC Guidelines for the diagnosis and treatment of acute and chronic heart failure: The Task Force for the diagnosis and treatment of acute and chronic heart failure of the European Society of Cardiology (ESC)Developed with the special contribution of the Heart Failure Association (HFA) of the ESC. Eur Heart J. 2016 Jul 14;37(27):2129–200.

4. Heuschmann PU, Montellano FA, Ungethüm K, Rücker V, Wiedmann S, Mackenrodt D, et al. Prevalence and determinants of systolic and diastolic cardiac dysfunction and heart failure in acute ischemic stroke patients: The SICFAIL study. ESC Heart Fail. 2021 Apr;8(2):1117–29.

5. Levey AS, Stevens LA, Schmid CH, Zhang YL, Castro AF, Feldman HI, et al. A new equation to estimate glomerular filtration rate. Ann Intern Med. 2009 May 5;150(9):604–12.

6. Geiger J, Both S, Kircher S, Neumann M, Rosenwald A, Jahns R. Hospital-integrated Biobanking as a Service – The Interdisciplinary Bank of Biomaterials and Data Wuerzburg (ibdw). 2018 Apr 23;5(0):6.

7. Ulm L, Hoffmann S, Nabavi D, Hermans M, Mackert BM, Hamilton F, et al. The Randomized Controlled STRAWINSKI Trial: Procalcitonin-Guided Antibiotic Therapy after Stroke. Front Neurol. 2017;8:153.

8. Hoffmann S, Harms H, Ulm L, Nabavi DG, Mackert BM, Schmehl I, et al. Stroke-induced immunodepression and dysphagia independently predict stroke-associated pneumonia - The PREDICT study. J Cereb Blood Flow Metab. 2017 Dec;37(12):3671–82.
